# Body Mass Index and Height in Friedreich Ataxia: What Do We Know?

**DOI:** 10.1212/NXG.0000000000000637

**Published:** 2021-11-12

**Authors:** Sylvia M. Boesch, Elisabetta Indelicato

**Affiliations:** From the Department of Neurology, Center for Rare Movement Disorders Innsbruck, Medical University Innsbruck, Austria.

Friedreich ataxia (FRDA) is a recessive disorder predominantly beginning in childhood or adolescence that causes progressive ataxia and dysarthria along with loss of sensation and coordination.^1^ Cardiomyopathy, scoliosis, diabetes, sensorineural hearing loss, and optic neuropathy are further manifestation of the disease. FRDA is caused by biallelic mutations in the FXN gene, which codes for the protein frataxin. The most common mutation is a biallelic expansion of a naturally occurring guanine-adenine-adenine (GAA) repeat in intron 1 (96%), while the remaining 4% of individuals carry 1 expansion along with a point mutation or a deletion. All disease-causing genotypes decrease, but not totally deplete, functional frataxin. FRDA is therefore primarily a disease of frataxin deficiency. Frataxin carries a mitochondrial targeting sequence, and its relative absence leads to the dysfunction of multiple mitochondrial processes. From a mechanistic point of view, all disease manifestations in FRDA reflect dysfunctions downstream of frataxin deficiency and reflect a primary mitochondrial pathology.

Recent evidence highlighted a relevant role of mitochondria in the imprinting of complex traits such as weight gain and growth.^2^ Pereira et al.^3^ reported that in nonhuman primates, maternal nutrient reduction (MNR) affects fetal cardiac left ventricular (LV) mitochondria. MNR caused a 2-fold increase in fetal LV mitochondrial DNA copy number in female fetuses but not in male fetuses. Several mitochondrial transcripts were differentially expressed between control and MNR fetuses. LV mitochondrial complex I and II/III activities and LV adenosine-triphosphate content were decreased by 73% in MNR fetuses, mostly in male fetuses. These findings suggest that the detrimental effect of MRN on fetal development may be substantially mediated by mitochondrial reprogramming in a sex-dependent fashion. In general, mitochondria are anticipated to affect genomic DNA methylation, histone acetylation, and phosphorylation via either coupling of the folate and methionine cycles or alterations in tricarboxylic acid cycle metabolites. Increased radical oxygen species and decreased adenosine-triphosphate as encountered in the setting of a mitochondrial dysfunction, are likely to influence epigenetic processes as well. Mitochondrial dysfunction in turn induces changes in the expression of mitochondrial genes that can further exacerbate both the dysfunction and aberrant epigenetic imprinting.

In this issue of *Neurology*® *Genetics*, members of the Friedreich Ataxia Clinical Outcome Measure Study (FACOMS) present a first large study that aimed to investigate cross-sectional and longitudinal associations between demographic, genetic, and clinical factors and anthropometric outcomes such as height and body mass index (BMI) in FRDA.^4^ The authors reported on 961 FRDA participants from 12 centers in the United States and Australia with a median age at baseline of 20 years, and 49% (n = 475) patients were female.

In children, 17% were underweight (BMI-Z <5th percentile) and only 8% were overweight or obese (BMI ≥85th percentile). Moreover, female sex in children was associated with lower BMI-Z. The aforementioned prevalence of underweight was therefore more than 5 times higher in children with FRDA and overweight/obesity around 2.5 times lower than a similarly aged pediatric US reference cohort (Association AH. CHME Data Visualizations 2017). Linear growth, including pubertal timing and velocity, was abnormal in this FRDA cohort. Gender differences in FRDA were detected regarding pubertal growth onset and spurt. In girls, the magnitude of the pubertal growth spurt was less, while in boys, it occurred later than in a healthy reference cohort. Until to date sex differences are not consistently reported in FRDA, but at least 1 earlier study reported a worse outcome in women with FRDA, with faster progression to mobility aids compared with men.^5^ The authors speculated that a lower BMI-Z score related to lower relative amounts of lean muscle mass in women might explain a more rapid functional decline and higher modified Friedreich Ataxia Rating Scale (mFARS) scores. Of interest in analyses restricted to children, the authors did not detect an association among GAA1 repeat length, age at symptom onset, or mFARS and BMI.

In adults, 7% of participants were found to be underweight (BMI < 18.5 kg/m^2^) and 33% overweight or obese (BMI ≥ 25 kg/m^2^). One possible cause for excess weight gain with advancing age is that because individuals with FRDA lose mobility over time, physical activity decreases. Moreover, excess weight may interfere with physical activity leading to a vicious circle. Supporting this potential explanation, in adults with other disorders affecting mitochondria, physical activity is inversely associated with BMI.^6^ Fatigue is commonly reported in individuals with FRDA and may further exacerbate inactivity and an associated increase in BMI over time. Moreover, in individuals without FRDA, BMI tends to increase from young adulthood (age 20–39 years) to middle adulthood (age 40–59 years). Therefore, the increase in BMI with advancing age in adults with FRDA may also reflect the natural history of BMI during lifetime. In adults, but not in children, genetic severity of disease, defined by GAA1 repeat length, and earlier age at FRDA symptom onset were associated with shorter stature. Shorter statue was also found in adults with other genetic mitochondrial disorders, strengthening height as a biomarker of disease burden in mitochondrial diseases.^7^

A limitation of this study is missing recordings of height and weight measurements in approximately a quarter of patients with FRDA (adults and children), especially in those with higher mFARS scores and/or nonambulatory patients. Another, even more relevant, limitation is the lack of assessment of general risk factors for inadequate growth and development. Apart from mutations in the FNX gene as a major genetic denominator, there are a bunch of factors known to affect growth and development: socioeconomic factors, family characteristics, human-made environment, nutrition, or even experiences during early childhood.^8^ Developmental plasticity, defined as the potential of a specific genotype to bring out diversified phenotypes in response to diverse environmental factors, may therefore have a major effect on further development in health and in disease.

It will be important to confirm current findings in future studies that include prospective collection of anthropometric measurements across the spectrum of disease severity and across the lifespan in FRDA. The introduction of assessment tools that address general risk factors for inadequate growth and development will add further valuable information apart from pure genetic burden. Understanding the relationship between weight gain, growth, as well as genetic and clinical characteristics in FRDA are prone to affect both clinical care and research investigations in FRDA in the future.
